# Mast cell marker gene signature in head and neck squamous cell carcinoma

**DOI:** 10.1186/s12885-022-09673-3

**Published:** 2022-05-24

**Authors:** Zhimou Cai, Bingjie Tang, Lin Chen, Wenbin Lei

**Affiliations:** Department of Otolaryngology, The First Affiliated Hospital of Sun Yat-sen University, No. 58 Zhongshan Er Road, Guangzhou, 510080 China

**Keywords:** Single-cell RNA sequencing, Head and neck squamous cell carcinoma, Tumour microenvironment, Immune infiltration, Risk score, Immunotherapy

## Abstract

**Background:**

Mast cells can reshape the tumour immune microenvironment and greatly affect tumour occurrence and development. However, mast cell gene prognostic and predictive value in head and neck squamous cell carcinoma (HNSCC) remains unclear. This study was conducted to identify and establish a prognostic mast cell gene signature (MCS) for assessing the prognosis and immunotherapy response of patients with HNSCC.

**Methods:**

Mast cell marker genes in HNSCC were identified using single-cell RNA sequencing analysis. A dataset from The Cancer Genome Atlas was divided into a training cohort to construct the MCS model and a testing cohort to validate the model. Fluorescence in-situ hybridisation was used to evaluate the MCS model gene expression in tissue sections from patients with HNSCC who had been treated with programmed cell death-1 inhibitors and further validate the MCS.

**Results:**

A prognostic MCS comprising nine genes (*KIT*, *RAB32*, *CATSPER1*, *SMYD3*, *LINC00996*, *SOCS1*, *AP2M1*, *LAT*, and *HSP90B1*) was generated by comprehensively analysing clinical features and 47 mast cell-related genes. The MCS effectively distinguished survival outcomes across the training, testing, and entire cohorts as an independent prognostic factor. Furthermore, we identified patients with favourable immune cell infiltration status and immunotherapy responses. Fluorescence in-situ hybridisation supported the MCS immunotherapy response of patients with HNSCC prediction, showing increased high-risk gene expression and reduced low-risk gene expression in immunotherapy-insensitive patients.

**Conclusions:**

Our MCS provides insight into the roles of mast cells in HNSCC prognosis and may have applications as an immunotherapy response predictive indicator in patients with HNSCC and a reference for immunotherapy decision-making.

**Supplementary Information:**

The online version contains supplementary material available at 10.1186/s12885-022-09673-3.

## Background

Tumour cells, blood vessels, immune cells, extracellular matrix, stromal cells, fibroblasts, pericytes, adipocytes, and various signalling factors function together to shape the tumour microenvironment (TME) [1]. Tumour cells can communicate with other types of cells in the TME [2]. As the major components of the TME, tumour-infiltrating immune cells exhibit cross talk with tumour cells to promote or suppress tumour growth, invasion, and metastasis [3]. Mast cells (MCs) are early and persistent tumour-infiltrating immune cells localised at the margins of tumours, most commonly around the blood vessels [4, 5]. Although most previous studies of MCs have focused on allergies, their ability to mediate tumour development and angiogenesis has been increasingly recognised [4]. Indeed, several studies have reported that MCs play a multifaceted role in modulating various events within the tumour [5]. MC infiltration within the TME is ubiquitous across various human cancers, and their accumulation has been associated with both pro- and antitumourigenic properties [6]. Thus, MCs are critical components of the TME and affect tumour prognosis, revealing their potential as therapeutic targets for cancer immunotherapy [6].

Head and neck squamous cell carcinoma (HNSCC) is the sixth most prevalent malignant tumour worldwide and primarily originates in the upper respiratory and digestive tracts, most commonly in the oral cavity, oropharynx, larynx, and hypopharynx, with an annual incidence of 900,000 cases and associated mortality of 450,000 deaths each year [7, 8]. Molecular changes in the parenchyma and complex, dynamic TME contribute to the wide heterogeneity of HNSCC, leading to differences in the growth rate, invasiveness, drug sensitivity, and prognosis among HNSCC tumours and thereby complicating treatment [9, 10]. Immune checkpoint inhibitor (ICI) therapy has recently gained attention as a promising therapeutic approach for HNSCC [11]. However, the therapeutic efficacy of ICI varies greatly among patients. For example, the response rate to programmed cell death-1 (PD-1)/PD-1 ligand (PD-L1) inhibitors for recurrent or metastatic HNSCC was only 13.3–17.9% in previous clinical trials [12, 13]. Differences between tumours determine the appropriate treatment modality; thus, characterising tumours is essential for providing precise treatment and improving the prognosis of patients with HNSCC. Precise population screening is an important strategy for improving the therapeutic efficacy of ICIs, which requires identification of more accurate molecular biomarkers to evaluate the tumour immune status, predict the treatment response, and perform risk stratification. Compared with normal tissues, the TME in HNSCC contains more MCs [14]. However, the role of MCs in the TME is complex and poorly understood. Some studies have found that the increased density of MCs in HNSCC is significantly associated with a reduced disease recurrence, and that small numbers of MCs may indicate the need for adjuvant therapy [15]. Alternatively, contrary findings suggest that increased mast cell density is closely associated with HNSCC angiogenesis and lymphatic vessel density, and may contribute to tumour progression [16]. Therefore, more sensitive MC-associated biomarkers must be identified, although the potential applications of MC-specific gene expression signatures have remained largely unexplored.

The emergence of single-cell RNA sequencing (scRNA-seq) technology has enabled analysis of cell types and transitions based on gene expression within tumours [17]. This technology can reveal the expression profile of single cells, thus identifying rare and previously undetected subpopulations within the tissue [18]. Therefore, scRNA-seq is valuable for studying cell populations and subpopulations within the TME. Cillo et al. [19] previously analysed the status of tumour-infiltrating immune cells in untreated HNSCC via scRNA-seq, revealing the full immune landscape of HNSCC and providing a reference dataset for in-depth studies of the roles of immune cells in HNSCC and other tumour types.

In the current study, we used this single-cell sequencing dataset (GSE139324) to comprehensively investigate the expression of MC characteristic genes (MCGs) in HNSCC and established an MCG-based prognostic marker for HNSCC for predicting the prognosis of both conventional and immunotherapy treatment.

## Materials and Methods

### Data acquisition

The Gene Expression Omnibus database (http://www.ncbi.nlm.nih.gov/geo/) was used to obtain scRNA-seq data from 60,976 intratumoural immune cells from 26 human primary HNSCC samples (accession number GSE139324) [19]. Bulk RNA-seq data for 501 HNSCC samples and 44 normal or paraneoplastic samples, as well as clinical and follow-up information for patients with HNSCC, were obtained by searching The Cancer Genome Atlas (TCGA; https://portal.gdc.cancer.gov/). Data from patients with missing survival times or survival times of fewer than 30 days were excluded from the current study because the patients may have died from other acute lethal conditions rather than from HNSCC.

### Processing of HNSCC scRNA-seq data and MCG identification

In total, 60,976 tumour-infiltrating immune cells from HNSCC were screened. The Seurat package in the R software (version 4.0.3; The R Project for Statistical Computing, Vienna, Austria) was utilised to analyse the scRNA-seq data [20]. First, cells with less than 500 detected genes or with more than 5% mitochondrial genes were considered low quality and removed. Cells with over 2500 genes were also filtered out to avoid doublets. After log-normalisation of the gene expression data, the top 2500 highly variable genes were screened using principal component analysis (PCA) to minimise the dimensionality of the scRNA-seq dataset [21]. The top 30 PCs were selected for dimensionality reduction, and major cell clusters were identified using the FindClusters function with a resolution of 0.6; the data were visualised using the t-distributed statistical neighbour embedding method [22]. Next, cluster-specific genes in each cluster were identified using the Findallmarker function. The cut-off criteria for marker gene identification were a false discovery rate (FDR) < 0.05 and |log2 fold-change| > 0.4. The “singleR” package was used to determine and annotate different cell clusters based on the composition of cluster-specific genes, which were then validated and corrected using marker genes provided by the CellMarker database [23, 24]. Finally, 51,127 cells were clustered into six major immune cell types. In TCGA-HNSCC dataset, the “limma” R package was used to identify differentially expressed MC cluster marker genes in tumour tissues and adjacent nontumour tissues, MC cluster marker genes with an FDR < 0.05 and |log2 fold-change| > 1 were defined as MCGs.

### Generation and validation of a prognostic signature based on MCGs

The patients were divided into training and test cohorts at a 7:3 ratio using the “caret” R package. First, in the training cohort, univariate Cox regression analysis of overall survival (OS) was performed on MCGs; those with a *P* value < 0.1 were considered to be related to the prognosis of HNSCC. Subsequently, to avoid overfitting when establishing the prognostic risk model, prognosis-related MCGs were subjected to LASSO Cox regression analysis, and the optimum penalty parameter (λ) value was used to generate the MCS [25]. Finally, the normalised expression level of each gene (*genei*) and its corresponding regression coefficients (*Expi*) were used to compute each patient’s risk score. The risk score was calculated using Eq. 1:

1 $$Risk\ score={\sum}_{i=1}^n Coef\ (genei)\ast Expi$$

Patients with HNSCC were divided into high- and low-risk groups based on median risk score values. Next, the Kaplan–Meier method was used to compare OS between high- and low-risk groups, and statistical differences were tested using log-rank tests. Univariate and multifactorial Cox regression analyses were performed to determine the prognostic value of MCS and patient clinicopathological variables. Furthermore, the time-dependent receiver operating characteristic (ROC) curves and area under the curve (AUC) values were calculated to validate the predictive accuracy of MCS and each clinical characteristic. To determine whether MCS could robustly differentiate patients, PCA was performed on patients according to the expression of MCS genes. The R packages used in the above steps included “stats,” “survival,” “survminer,” and “survROC.”

### Functional enrichment and molecular analyses between risk groups

Gene Ontology (GO) and Kyoto Encyclopedia of Genes and Genomes (KEGG) analyses of differentially expressed genes (DEGs, cut-off values: FDR < 0.05 and |log2 fold-change| > 1) between the low- and high-risk groups were performed using the “clusterProfiler” R package to determine the biological functions and pathways associated with the risk score [26].

Gene set enrichment analysis (GSEA) was used to identify subtle differences in each enriched KEGG pathway in the high- and low-risk groups [27].

### Immune cell infiltration and immune-related pathway analyses

Single-sample GSEA (ssGSEA) was used to estimate the level of immune cell infiltration and immune-related pathway activity among different risk groups [28].

### Roles of MCS in predicting immunotherapeutic benefits

We used the “limma” and “ggpubr” R packages to identify the relationships between the risk score and ICI response-related gene expression to predict which patient group may benefit from immunotherapy.

Because the immunophenoscore (IPS) is a superior predictor of the response to anti-cytotoxic T-lymphocyte antigen (CTLA)-4 and anti-PD-1 regimens, we further evaluated the role of MCS in predicting immunotherapy response by comparing the relationships between IPS and different risk groups. For this analysis, IPSs of patients with HNSCC were obtained from The Cancer Immunome Database (https://tcia.at/home) [29].

### Verification of model genes using double-label fluorescence in-situ hybridisation (FISH)

FISH was performed to detect the expression of model genes in tissue sections from patients with HNSCC who had been treated with PD-1 inhibitors (treatment effects are shown in Table S1). Cy3-labeled (red) probes specific to high-risk genes and FAM-labelled (green) probes specific to low-risk genes were designed and synthesised by Servicebio (Wuhan, China). Briefly, prehybridisation buffer was added to unstained tissue sections and incubated at 37 °C for 60 min. The first probe hybridisation solution was added to each section and incubated overnight at 42 °C. Excess hybridisation solution was removed by washing, and mouse anti-digoxigenin-labelled horseradish peroxidase was then added and incubated at 37 °C for 40 min. The sections were dried, and freshly prepared TSA chromogenic reagent was added to the labelled tissue. The sections were incubated with fluorescein-labelled secondary probe hybridisation solution for 3 h. Nuclei were counterstained with 4′,6-diamidino-2-phenylindole (DAPI) in the dark for 8 min. Two corresponding excitation filters were selected and observed under a fluorescence microscope to locate and quantify the two genes. To better show the expression of high- and low-risk genes, double-labelled genes were used to select high- and low-risk gene pairs, respectively. The probe sequences used in this study are listed in Table S2.

### Ethical statement

All study designs and test procedures were performed in accordance with the Helsinki Declaration II. The study was approved by the ethics board of IEC for Clinical Research and Animal Trials of the First Affiliated Hospital of Sun Yat-sen University (approval no. [2020]220-1). All patients in this study signed informed consent and agreed to follow up after treatment.

### Statistical analysis

All statistical analyses were performed using the R software (version 4.0.3). All differences were considered as statistically significant when the *P* value was < 0.05, and all tests were two-tailed.

## Results

### Identification of MC cluster marker genes and MCGs

**Fig. 1:**
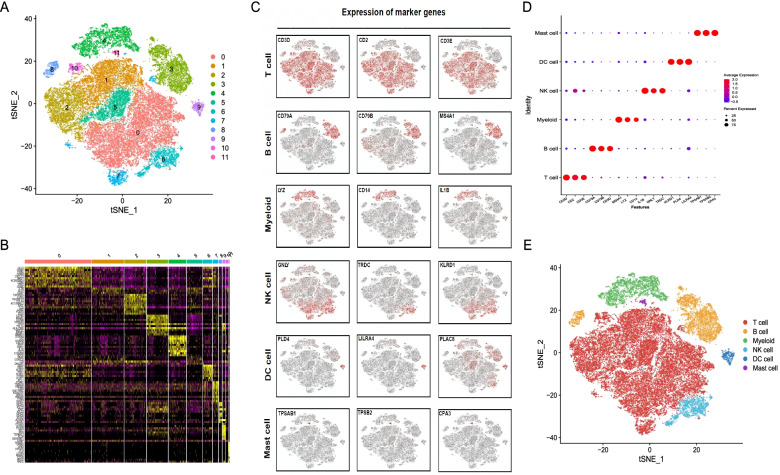
Single-cell RNA sequencing analysis to identify marker genes in mast cells. **A** t-Stochastic neighbour embedding (t-SNE) plots for immune cells. **B** Heat map showing the expression levels of specific marker genes in each cluster. **C** t-SNE plots displaying representative marker gene expression levels for six cell types. **D** Bubble plots showing the expression of marker genes in six cell types. **E** t-SNE plots showing cell types among 51,127 immune cells

After quality control (Fig. S1), 51,127 cells were clustered into 12 major clusters (Fig. 1A and B). Cluster-specific genes were determined. The “singleR” R package and CellMarker database were used to annotate the clusters into six types of immune cells: T cells (CD3D^+^), natural killer cells (GNLY^+^), B cells (CD79A^+^), dendritic cells (PLD4^+^), myeloid cells (LYZ^+^), and MCs (TPSAB1^+^; Fig. 1C–E). In the TCGA-HNSCC dataset, we analysed the differential expression of MC cluster marker genes obtained from the GSE139324 dataset in 501 tumour and 44 adjacent nontumour tissues, and 47 DEGs (MCGs) were identified (FDR < 0.01). The heat and volcano maps in Figs. S2 and S3 display the transcript levels of these genes.

### Independent prognostic value of the MCS risk model

Univariate and multivariate Cox regression analyses of clinicopathological variables (age, sex, tumour grade, and tumour stage) and overall survival revealed that the MCS risk score could be used as an independent predictor of patient prognosis in the training (Fig. 4A, B), testing (Fig. 4D, E), and entire TCGA cohorts (Fig. 4G, H; *P* < 0.05). In addition, the multi-indicator ROC curve showed that the AUCs in these cohorts were 0.699 (Fig. 4C), 0.682 (Fig. 4F), and 0.692 (Fig. 4I), respectively, suggesting that our prognostic model was superior for predicting patient outcome relative to the remaining clinical indicators.

### Construction and validation of prognostic MCS

HNSCC samples meeting the screening criteria (*n* = 490) were randomly divided into training (*n* = 346) and test (*n* = 144) cohorts at a ratio of 7:3. Table 1 shows the clinical characteristics of patients with HNSCC in the different cohorts. In the training cohort, 14 MCGs associated with prognosis were identified utilising univariate Cox regression analysis. The 14 candidate genes were narrowed down using LASSO Cox regression, and a nine-gene signature (MCS) was established based on the best λ value (Fig. S2). Detailed information and coefficients of the nine genes are presented in Table 2 and Table S3. The risk score was calculated as follows: risk score (MCS) = (− 0.0355 × *KIT* expression) + (0.0018 × *RAB32* expression) + (0.1094 × *CATSPER1* expression) + (0.0233 × *SMYD3* expression) + (− 0.4625 × *LINC00996* expression) + (− 0.0168 × *SOCS1* expression) + (0.0007 × *AP2M1* expression) + (− 0.7264 × *LAT* expression) + (0.0015 × *HSP90B1* expression). The MCS of each patient was calculated, and patients in each cohort were split into low- and high-risk subgroups based on the median risk score (1.1413) obtained from the training cohort.

**Table 1:** Clinical parameters of HNSCCs patients in the TCGA databases. Clinical parameters

Clinical Pareameters	Training cohort		Testing cohort		Entire TCGA cohort	
	***n =*** 346	%	***n =*** 144	%	***n =*** 490	%
**Age**						
≤ 65	224	64.74	97	67.36	321	65.51
>65	122	35.26	47	32.64	169	34.49
**Sex**						
Female	96	27.75	34	23.61	130	26.53
Male	250	72.25	110	76.39	360	73.47
**Histologic grade**						
G1-2	252	72.83	101	70.14	353	72.04
G3-4	83	23.99	35	24.31	118	24.08
GX	11	3.18	5	3.47	16	3.27
NA	0	0	3	2.08	3	0.61
**T classification**						
T1-2	129	37.28	45	31.25	174	35.51
T3-4	179	51.73	82	56.94	261	53.27
TX	24	6.94	9	6.25	33	6.73
NA	14	1.05	8	5.6	22	4.49
**N classification**						
N0	121	34.97	45	31.25	166	33.88
N+	160	46.24	71	49.31	231	47.14
NX	50	14.45	19	13.19	69	14.08
NA	15	4.34	9	3.25	24	4.90
**M classification**						
M0	120	34.68	60	41.67	180	36.73
M1	1	0.29	0	0	1	0.21
MX	44	12.72	16	11.11	60	12.24
NA	181	52.31	68	47.22	249	50.82
**Stage**						
I-II	70	20.23	24	16.67	94	19.18
III-IV	228	65.90	100	69.44	328	66.94
NA	48	13.87	20	13.89	68	13.88
**Vital status**						
Deceased	144	41.62	67	46.53	211	43.06
Living	202	58.38	77	53.47	279	56.94

**Table 2:** List of the nine Mast cell signature genes of the MCS in HNSCC

ENSG ID	Symbol	Location	Expression status	Coefficient
ENSG00000157404	KIT	Chr4: 54657918-54,740,715	Down	−0.0355
ENSG00000118508	RAB32	Chr6: 146543833-146,554,953	Up	0.0018
ENSG00000175294	CATSPER1	Chr11: 65784223-65,793,950	Up	0.1094
ENSG00000185420	SMYD3	Chr1: 245749342-246,507,312	Up	0.0233
ENSG00000242258	LINC00996	Chr7: 150130742-150,145,228	Up	− 0.4625
ENSG00000185338	SOCS1	Chr16: 11348274-11,350,039	Up	− 0.0168
ENSG00000161203	AP2M1	Chr3: 184174689-184,184,091	Up	0.0007
ENSG00000213658	LAT	Chr16: 28984803-28,990,784	Up	−0.7264
ENSG00000166598	HSP90B1	Chr12: 103930107-103,953,931	Up	0.0015

**Fig. 2:**
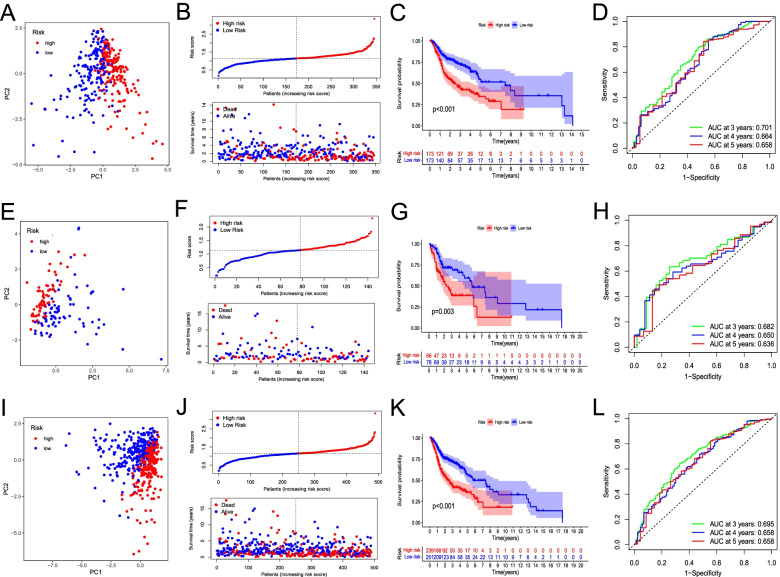
Prognostic analysis of the mast cell gene signature (MCS) risk score. PCA plot (**A, E, I**); risk score analysis (**B, F, J**); Kaplan–Meier curve survival analysis (**C, G, K**); time-receiver operating characteristic curve analysis (**D, H, L**) in the training, testing, and entire “The Cancer Genome Atlas” cohorts, respectively

The PCA results indicated that patients in each cohort were clearly separated into two clusters based on MCS (Fig. 2A, E, I). In the training cohort, higher risk scores corresponded to an increased patient mortality rate (Fig. 2B). Consistent with this result, the Kaplan–Meier curve indicated a significantly shorter OS for patients in the high-risk group compared with those in the low-risk group (Fig. 2C; *P* < 0.001). The sensitivity and specificity of the MCS prognostic model were evaluated using ROC analysis, and the AUC values for the 3-, 4-, and 5-year OS were 0.701, 0.664, and 0.658, respectively (Fig. 2D). The testing and entire TCGA cohorts showed similar results as the training cohort. The mortality rate of patients increased with the risk score (Fig. 2F, J), and high-risk patients showed a shorter OS (Fig. 2G, K). In addition, the 3-, 4-, and 5-year OS AUC values were all greater than 0.65 (Fig. 2H, L). These results support the reliability of the MCS prognostic model.

**Fig. 3:**
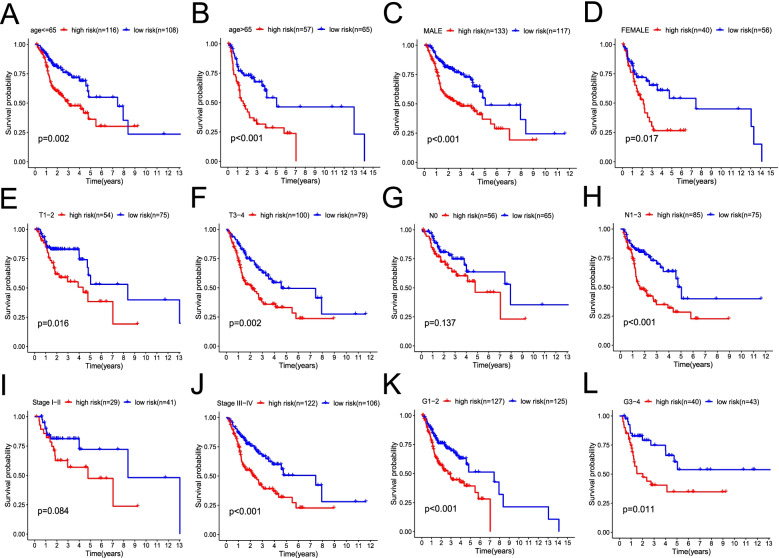
Prognostic value of the mast cell gene signature (MCS) risk score in the training cohort classified based on clinicopathological variables. Survival curve between high- and low-risk groups stratified by ages (**A, B**); sex (**C, D**); T stage (**E, F**); N stage (**G, H**); stage (**I, J**); and grade (**K, L**)

To determine the prognostic potential of the MCS model in the clinical setting, patients were grouped according to clinicopathological variables (age, sex, T stage, N stage, stage, and grade). For patients of different ages (≤65 years old [young group] versus > 65 years old [old group]), sexes, T stages, and grade groups and those in the N1-3 group and stage III–IV group, the OS rates of patients rated as high-risk by MCS were significantly reduced (all *P* < 0.05). However, for patients with N0 and stage I/II disease, no significant differences were observed, possibly because of the small sample size (Fig. 3). In the testing and entire TCGA cohort, after grouping by clinicopathological variables, the survival results following MCS-based risk stratification were similar to those in the training group (Fig. S4).

**Fig. 4:**
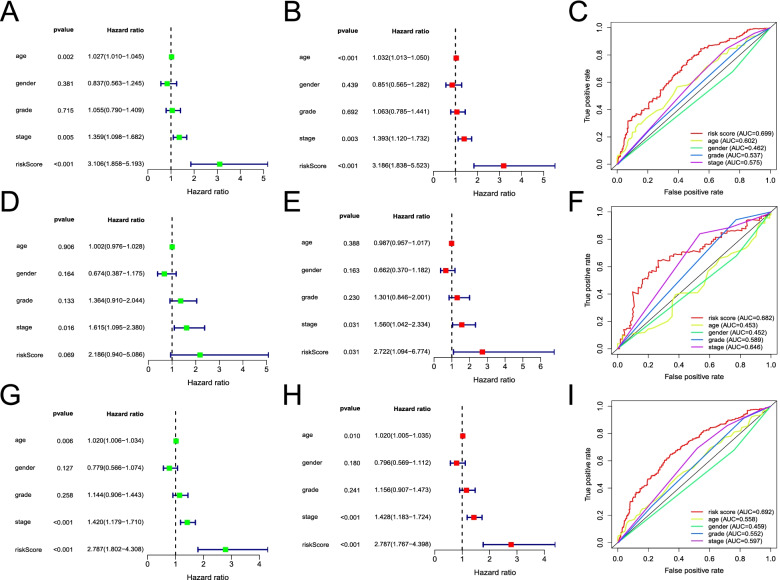
Predictive effects of the mast cell gene signature (MCS) risk score and clinicopathological variables on the prognosis of overall survival of patients with head and neck squamous cell carcinoma. Univariate and multivariate Cox regression analyses between clinicopathological variables (including the MCS risk score) and overall survival of patients in the training (**A, B**), testing (**D, E**), and the entire “The Cancer Genome Atlas” (**G, H**) cohorts; green and red squares represent univariate and multivariate analysis, respectively. Comparison of area under the receiver operating characteristic curve between clinicopathological variables and MCS risk score in the training, testing, and the entire “The Cancer Genome Atlas” cohorts (**C, F, I**, respectively)

**Fig. 5:**
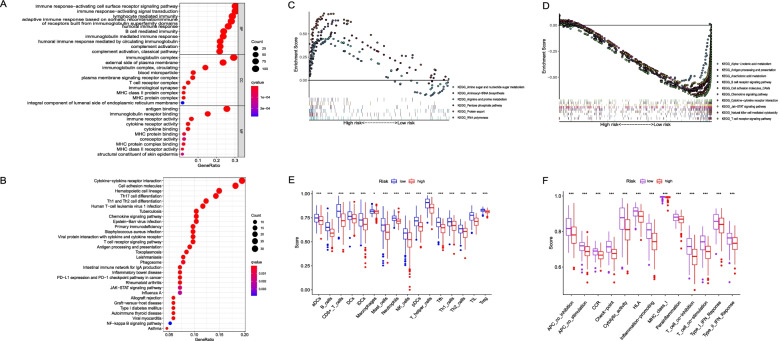
Functional and molecular characteristics analysis of the high- and low-risk groups in the entire “The Cancer Genome Atlas” cohort. **A** Bubble graph for Gene Ontology enrichment (a larger bubble indicates more enriched genes, and an increasing depth of red indicates greater differences; q-value: adjusted *P* value; GeneRatio: number of DEGs annotated to the GO or KEGG pathway/total number of DEGs). **B** Bubble graph for the Kyoto Encyclopedia of Genes and Genomes pathways. **C** Multiple gene set enrichment analysis showing the enriched pathways of the high-risk and (D) low-risk subgroups. **E** Boxplots show the comparison of single-set gene set enrichment analysis scores for 16 immune cell types and **F** 13 immune-related functions. CCR, cytokine-cytokine receptor. Adjusted *P* values are shown as: *P < 0.05; ***P < 0.001

**Fig. 6:**
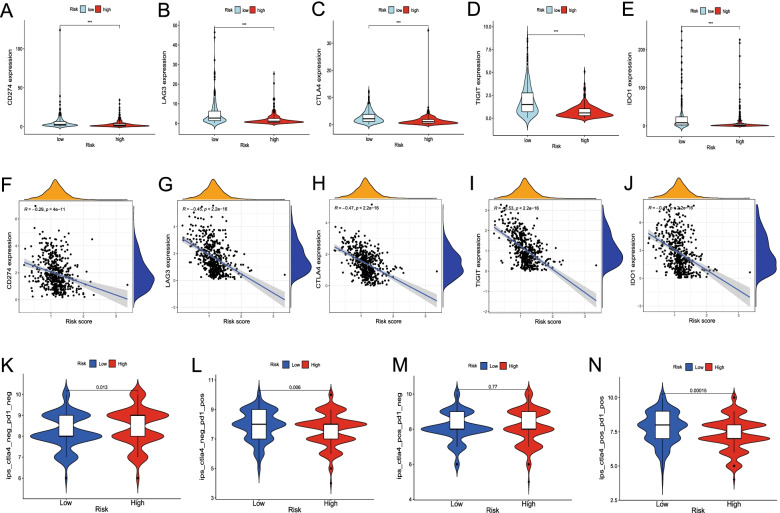
Estimation of the roles of the mast cell gene signature (MCS) in predicting immune checkpoint gene expression and immunotherapeutic response. Expression of immune checkpoint genes in different risk groups of the training cohort, violin plot of *CD274* (*PD-L1*) (**A**), *LAG3* (**B**), *CTLA4* (**C**), *TIGIT* (**D**), and *IDO1* (**E**) expression in the low- and high-risk groups. ***P < 0.001. Correlation between the risk scores and immune checkpoint gene expression, scatter plot of *CD274* (*PD-L1*) (**F**), *LAG3* (**G**), *CTLA4* (**H**), *TIGIT* (**I**), and *IDO1* (**J**) expression with risk scores. Association between the immunophenoscore and MCS in patients with head and neck squamous cell carcinoma (HNSCC) based on The Cancer Immunome Database CTLA-4^−^PD-1^−^ (**K**), CTLA-4^−^PD-1^+^ (**L**), CTLA-4^+^PD-1^−^ (**M**), CTLA-4^+^PD-1^+^ (L)

**Fig. 7:**
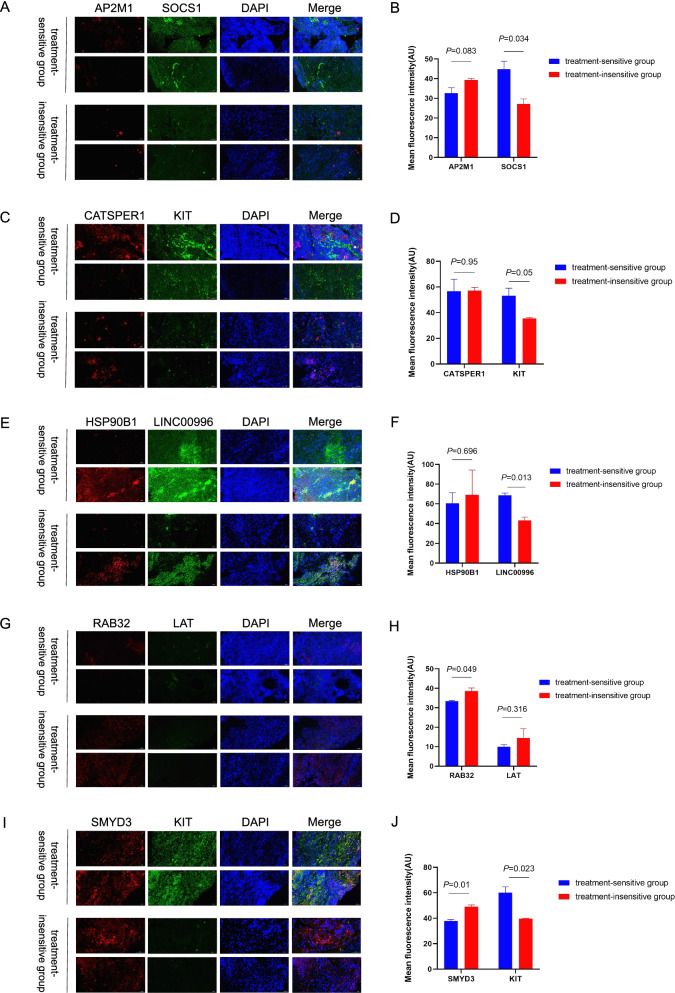
(**A, C, E, G, I**) Fluorescence in-situ hybridisation (FISH) assay was conducted to determine the expression of model genes in the low-risk and high-risk groups. Nuclei are stained blue (DAPI), and AP2M1, CATSPER1, HSP90B1, RAB32, SMYD3 are stained red. SOCS1, KIT, LINC00996, and LAT are stained green. Scale bar, 50 μm. (**B, D, F, H, J**) ImageJ was used to measure the mean fluorescence intensity of each gene staining in the images, and the t-test was used to analyse the intergroup significance

### Functional analyses and molecular characteristics of different MCS risk groups

As shown in Fig. 5, the DEGs extracted by the high- and low-risk groups in the entire TCGA cohort were used to perform GO enrichment and KEGG pathway analyses. As expected, the DEGs were associated with the immune response and cell-mediated immunity, indicating that MCs induced inflammatory responses within the TME. The cytokine-cytokine receptor interaction pathway was the most significantly enriched KEGG pathway, whereas other DEGs were predominantly enriched in cell adhesion molecules (CAMs) and chemokine signalling, T cell receptor signalling, Janus kinase (JAK)/signal transducer and activator of transcription (STAT) signalling, and multiple T-cell differentiation pathways. The GO and KEGG enrichment results indicated that MCs could regulate the composition of and immune response within the TME. Similar GO and KEGG enrichment results were observed in the training and testing cohorts (Fig. S5).

GSEA revealed 15 and 66 KEGG pathways significantly active in the high- and low-risk groups, respectively (FDR < 0.25 and nominal *P* < 0.05), and the top six and ten pathways with the highest normalised enrichment score in the high- and low-risk groups were chosen for visualisation analysis (Table 3). The high-risk group had higher enrichment levels of amino sugar and nucleotide sugar metabolism, aminoacyl-tRNA biosynthesis, and arginine and proline metabolism (Fig. 5C). In the low-risk group, the alpha-linolenic acid and arachidonic acid metabolism pathways were significantly enriched (Fig. 5D). Notably, CAMs, natural killer cell-mediated cytotoxicity, B cells, and T-cell receptor signalling pathways were obviously enriched in the low-risk group but attenuated in the high-risk group (Fig. 5D).

**Table 3:** The ten representative KEGG pathways in high- and low-risk groups

Names	Size	ES	NES	NOM *P*	FDR
High-risk group					
KEGG_Protein export	24	0.72	1.94	0.002	0.097
KEGG_Aminoacyl-tRNA biosynthesis	41	0.66	1.88	0.006	0.103
KEGG_RNA polymerase	28	0.64	1.84	0.004	0.092
KEGG_Pentose phosphate pathway	27	0.52	1.61	0.037	0.239
KEGG_Amino sugar and nucleotide sugar metabolism	43	0.46	1.58	0.032	0.224
KEGG_Arginine and proline metabolism	54	0.42	1.53	0.038	0.211
Low-risk group					
KEGG_T cell receptor signaling pathway	108	−0.7	−2.3	0	0
KEGG_Cytokine-cytokine receptor interaction	264	−0.63	−2.26	0	0
KEGG_Chemokine signaling pathway	188	−0.63	−2.23	0	0
KEGG_Natural killer cell mediated cytotoxicity	132	−0.63	−2.19	0	0
KEGG_Cell adhesion molecules_CAMs	131	−0.68	−2.19	0	0
KEGG_Jak-STAT signaling pathway	155	−0.6	−2.16	0	0.001
KEGG_B cell receptor signaling pathway	75	−0.65	−2.13	0	0.001
KEGG_Antigen processing and presentation	81	−0.68	−2.05	0	0.001
KEGG_Alpha-Linolenic acid metabolism	19	−0.7	−1.92	0	0.008
KEGG_Arachidonic acid metabolism	58	−0.55	−1.91	0	0.009

### Differences in immune cell infiltration and pathways between subgroups

Functional analyses revealed that the MCS was related to antitumour immunity. We further analysed the immune cells and immune-related pathways among different risk groups utilising ssGSEA and found that the high-risk group showed significantly less infiltration of all immune cells compared with the low-risk group (macrophages, *P* < 0.05; all other immune cells, *P* < 0.001; Fig. 5E). Accordingly, all 13 immune pathways exhibited significantly lower activity in the high-risk group than in the low-risk group (all *P* < 0.001; Fig. 5F). Assessment of the immune status in the testing and entire TCGA cohorts showed similar results (Fig. S6).

### Role of MCS in predicting immunotherapeutic benefits

KEGG pathway analysis indicated that PD-L1 expression and PD-1 checkpoint pathways in cancer were enriched in DEGs. Together with the previous analyses indicating that high- and low-risk patients had significant differences in immune cell infiltration, we examined whether the MCS was associated with ICI-related biomarker expression and could be used to predict immunotherapy benefits. In the training TCGA cohort, the high-risk group was positively correlated with low expression of *CD274* (PD-L1; *P* < 0.001, Fig. 6A), *LAG3* (*P* < 0.001, Fig. 6B), *CTLA4* (*P* < 0.001, Fig. 6C), *TIGIT* (*P* < 0.001, Fig. 6D), and *IDO1* (*P* < 0.001, Fig. 6E). The expression of these ICI-related marker genes decreased with increasing MCS risk scores (Fig. 6F–J).

As shown in Fig. 6K–L, patients with HNSCC in the training cohort could be divided into four types according to the expression of CTLA-4 and PD-1. In the CTLA-4^−^PD-1^−^, CTLA-4^−^PD-1^+^, and CTLA-4^+^PD-1^+^ groups, the IPS of low-risk patients was significantly higher than that of high-risk patients. A higher IPS was positively correlated with a better response to anti-CTLA-4 and anti-PD-1 treatment [29]. These results collectively suggested that the MCS could predict the immunotherapy response, with patients rated as low-risk by the MCS more likely to benefit from immunotherapy. The testing and entire TCGA cohort showed similar results (Fig. S7).

### Relative expression of MCS in the two groups of patients with HNSCC

Next, we used FISH to assess the expression patterns of nine model genes in tissue sections from HNSCC patients treated with PD-1 inhibitors. Representative images were obtained from four patients, two from the treatment-insensitive group and two from the treatment-sensitive group. The green fluorescent signal intensity was generally stronger in the treatment-sensitive group than in the treatment-insensitive group. Further analysis using Image J showed that the green fluorescence signal intensity of SOCS1, KIT, and LINC00996 in the treatment-sensitive group significantly differed from that of the treatment-insensitive group (Fig. 7A-F). In addition, the red fluorescence signal intensity of RAB32 and SMYD3 in the high-risk group was significantly higher than that of these genes in the treatment-sensitive group (Fig. 7G-J). The fluorescence signal intensities of AP2M1, CATSPER1, HSP90B1, and LAT, did not significantly differ between the two groups (Fig. 7A-H). The above results further verified the accuracy of the MCS risk model, that is, high-risk genes were highly expressed in the immunotherapy-insensitive group, while low-risk genes were highly expressed in the immunotherapy-sensitive group.

## Discussion

The degree of immune cell infiltration and activation within the TME divides tumours into two types: immunologically hot (inflamed) and cold (noninflamed) [30, 31]. Patients with the latter tumour type have poorer prognoses and benefit less from immunotherapy [32]. Therefore, analysing the abundance and types of tumour-infiltrating immune cells is essential for improving patient stratification and treatment outcome prediction. HNSCC malignancies tend to develop into immunologically cold tumours, compromising the response to immunotherapy [10, 33]. An increasing number of studies have reported that MCs play a protumourigenic role by stimulating tumour cell growth [34], inducing an immunosuppressive TME [35], promoting angiogenesis and lymphangiogenesis [36], and facilitating invasion and metastasis [37]. High MC numbers are associated with the poor clinical prognosis of various solid tumours, including colorectal [38], gastric [39], and pancreatic [40] cancers. However, the roles of MCs in these tumours remain controversial. The study of Kaesler et al. [41] pointed out that MCs is a biomarker for improving the survival rate of melanoma patients and believed that targeted activation of MCs can effectively promote T cell-mediated tumour cell clearance. Similarly, the study by Attranmadal et al. [15] showed that an increase in MCs density was significantly associated with a reduction in HNSCC recurrence, and further suggested that a small number of MCs might suggest the need for additional adjuvant therapy. In these previous studies, MC abundance was determined by observing tumour slices. Such approaches are limited for functionally distinguishing tumour-promoting and tumour-antagonizing MCs. To date, the roles of MCs in HNSCC remain unclear. To comprehensively analyze the expression patterns and prognostic significance of MCGs for HNSCC, we analyzed scRNA-seq and constructed an MCS risk signature reflecting the immune infiltration of HNSCC.

We constructed a novel prognostic signature integrating nine MCGs and validated its prognostic value using data from patients with HNSCC. According to our MCS model, high expression levels of *RAB32*, *CATSPER1*, *SMYD3*, *AP2M1*, and *HSP90B1* were associated with poor prognosis, whereas high expression levels of *KIT*, *LINC00996*, *SOCS1*, and *LAT* were associated with improved prognosis. *RAB32* is a member of the Ras proto-oncogene family that encodes an A-kinase anchoring protein [42]. A recent study demonstrated that RAB32 is a mechanistic target of the rapamycin complex 1 signaling pathway, and elimination of RAB32 has been shown to decrease tumour cell viability and proliferation [43]. SMYD3 promotes tumour cell migration and invasion by strengthening epithelial-mesenchymal cells and enhancing telomerase activity during the cell cycle [44–47]. Furthermore, SMYD3 expression is a key risk factor for esophageal [47], breast [46], bladder [48], and other [49] cancers. A recent study indicated that AP2M1 was overexpressed in adenoid cystic and mucoepidermoid carcinomas and could serve as a prognostic marker of hepatocellular carcinoma [50, 51]. Increased HSP90B1 expression has been shown to be an indicator of poor prognosis in patients with lung cancer [52], chronic lymphocytic leukemia [53], bladder cancer [54], and liver cancer [55]. Our findings, which are consistent with previous research, indicated that *RAB32*, *SMYD3*, *AP2M1*, and *HSP90B1* were associated with cancer progression. Few studies have focused on the roles of *CATSPER1* in tumours; in this study, we found that *CATSPER1* was related to HNSCC prognosis. Thus, further research on the molecular functions of *CATSPER1* in tumours is warranted. Regarding genes whose expression was associated with more favorable prognosis, *KIT* encodes a cell surface receptor for stem cell factors of the type III receptor tyrosine kinase family, with MCs among the main cell types expressing KIT [56]. KIT activation is important for normal cell development, growth, and differentiation [57]. However, gain-of-function mutations in the *KIT* gene can promote tumour formation and progression [58]. The molecular mechanism of *LINC00996* in tumours is unclear. Through data mining and bioinformatics, Ge et al. [59] suggested that decreased *LINC00996* expression is related to the occurrence and metastasis of colorectal cancer. In addition, they suggested that JAK/STAT, nuclear factor-κB, hypoxia-inducible factor-1, Toll-like receptor, and phosphatidylinositol 3-kinase/AKT signaling pathways are key pathways through which *LINC00996* suppresses tumourigenesis and metastasis. Thus, *LINC00996* should be further studied in the context of cancer. SOCS1 is the main regulator of various cytokines involved in the immune response, particularly the interferon-γ signaling pathway. Recent findings have suggested that SOCS1 is a tumour suppressor, and its downregulation has been implicated in cancer progression [60]. Furthermore, SOCS1 is silent in 50% of liver cancer cases [61], 44% of gastric cancer cases [62], 75% of melanoma cases [63], and 40% of hepatoblastoma primary tumours [64]. Linker for activation of T cells (LAT) is the nucleation site of the multiprotein signaling complex, which is essential for the function and differentiation of T cells; thus, its association with tumour prognosis is expected. Our current findings and those of previous studies suggest that the MCS may have applications in prognosis prediction for patients with HNSCC based on MCGs.

DEGs between the high- and low-risk groups were evaluated to determine the biological functions and pathways associated with the risk score. The DEGs were predominantly related to the immunological response and immune cell-mediated immunity, CAMs, and multiple T-cell differentiation pathways. Furthermore, we conducted GSEA to detect subtle expression changes between MCS groups. Unlike traditional enrichment analysis based on hypergeometric distribution, GSEA does not depend on individual gene expression changes but rather detects changes in the expression of gene sets. GSEA results revealed significant differences in immune function-related pathways between MCS-based risk groups. Specifically, compared with the low-risk group, the high-risk group was missing CAMs, natural killer cell-mediated cytotoxicity, as well as B- and T-cell receptor signaling pathways. Hence, significant differences in the immune environment were observed among the different MCS-based risk populations; patients in the high-risk group showed a significantly suppressed tumour immune microenvironment, and a high-risk score may be associated with attenuated natural killer cell cytotoxicity as well as B-cell and T-cell signaling.

ssGSEA further validated this idea. The results of ssGSEA showed that the infiltration level of 16 immune cells and the activity of 13 immune pathways in the high-risk group were significantly lower than those in the low-risk group. Notably, MCs were significantly less infiltrated in the high-risk group than in the low-risk group (*p* < 0.001), this finding is consistent with the study of the prognostic role of MCs in HNSCC by Attranmadal et al. [15], that higher MC infiltration was associated with better prognosis. It is worth mentioning that MCs have complex interactions with a variety of immune cells. Studies have shown that activated MCs can recruit tumour-infiltrating effector T cells and natural killer cells by secreting CXCL10 and CXCL8, respectively [41, 65]; in addition, mast cells can also greatly alter B cell generation, development, and function by secreting cytokines such as IL-6 [66]. This partly explains why the low-risk group with higher mast cell infiltration is enriched for natural killer cell-mediated cytotoxicity, as well as B- and T-cell receptor signaling pathways. Collectively, these findings indicate that a high MCS score is related to an immunosuppressive status and MCS may has the potential to predict the tumour immune microenvironment of HNSCC patients.

Recently, tumour immunotherapy has led to new opportunities for suppressing tumour progression, recurrence, and metastasis. Notably, immunotherapy is largely ineffective in immunologically cold tumours [67], including HNSCC tumours, which often acquire this characteristic. Thus, immunotherapy efficacy is compromised in cold HNSCC tumours with an objective response rate of single-agent anti-PD-1/PD-L1 immunotherapy as low as 13–14% in patients with HNSCC who are not screened for immune checkpoint expression prior to treatment [12, 68, 69]. Therefore, clinicians must consider the tumour immune status of patients with HNSCC prior to treatment selection. In contrast to cold tumours, hot tumours are characterised by considerable immune cell infiltration, particularly that of cytotoxic T cells, in addition to high expression of immune checkpoint molecules, such as PD-1, PD-L1, and LAG3 [30, 67]. The expression of these three factors is an important indicator of the immunotherapy response [70–72]. Immunohistochemistry and quantitative immunofluorescence are commonly used to detect immune checkpoint expression; improved methods are needed for this analysis [73]. We found that the DEGs were enriched in PD-L1 expression and PD-1 checkpoint pathways in cancer, suggesting that MCS could predict PD-L1 expression and thus immunotherapy responsiveness. Therefore, using the MCS model, we attempted to predict the potential benefit of immunotherapy for different risk groups. As expected, the expression of *PD-L1*, *LAG3*, *CTLA4*, *TIGIT*, and *IDO1* was significantly lower in the high-risk group than in the low-risk group. In addition, the IPS score was significantly higher in the low-risk group than in the high-risk group, which is an excellent predictor of the anti-CTLA-4 and anti-PD-1 response. Taken together with the results of immune cell infiltration, patients rated as low risk by MCS will benefit more from immunotherapy than those rated as high risk by MCS. Interestingly, the low-risk group showed a higher mutation frequency in *CDKN2A*; a recent study suggested that *CDKN2A/2B* mutation is related to high PD-1/PD-L1 expression and promotes the efficacy of immunotherapy, which is consistent with our findings [74].

Few studies have focused on MC characteristic genes in HNSCC, particularly their underlying mechanisms. We preliminarily explored the prognostic value of MC characteristic genes, providing a theoretical basis for future research. Additionally, our findings indicate that the MCS based on nine MCGs can be used to predict the immunotherapy response, thus providing information for the development of precision medicine approaches. However, a limitation of the study is that our findings cannot be confirmed through external verification. Nevertheless, the MCS was validated in multiple HNSCC cohorts. Furthermore, we validated our observations in clinical specimens. Taken together, more prospective data are needed to verify its clinical utility and to explore the mechanism of action of MCs in tumours.

In summary, we established a risk model based on nine MCGs to predict prognosis in patients with HNSCC and evaluate immune cell infiltration and the immune function status. Furthermore, the MCS can be utilised to screen patients suitable for immunotherapy and design optimal treatment strategies.

## Supplementary Information

**Additional file 1.**

## Data Availability

The datasets during and/or analysed during the current study are available from the corresponding author on reasonable request. Data from TCGA cohort and GEO are public.

## Abbreviations

TME: Tumour microenvironment
MC: Mast cell
ICI: Immune checkpoint inhibitor
HNSCC: Head and neck squamous cell carcinoma
PD-1: Programmed cell death-1
PD-L1: Programmed cell death-1 ligand
scRNA-seq: Single-cell RNA sequencing
MCG: Mast cell characteristic gene
TCGA: The Cancer Genome Atlas
PCA: Principal component analysis
FDR: False discovery rate
OS: Overall survival
ROS: Receiver operating characteristic
AUC: Area under the curve
GO: Gene Ontology
KEGG: Kyoto Encyclopedia of Genes and Genomes
DEG: Differentially expressed genes
GSEA: Gene set enrichment analysis
ssGSEA: Single-sample gene set enrichment analysis
IPS: Immunophenoscore
CTLA: Cytotoxic T-lymphocyte antigen
FISH: Fluorescence in situ hybridisation
DAPI: 4′,6-diamidino-2-phenylindole
CAM: Cell adhesion molecule
JAK: Janus kinase
STAT: Signal transducer and activator of transcription
LAT: Linker for activation of T cells
