# Supplementary material for: Mast cell marker gene signature in head and neck squamous cell carcinoma
Source: BMC Cancer. 2022 May 24;22:577. doi: 10.1186/s12885-022-09673-3 (PMC9128261; doi:10.1186/s12885-022-09673-3)
Supplement: Supplementary file 1 — Additional file 1. [file 12885_2022_9673_MOESM1_ESM.docx]

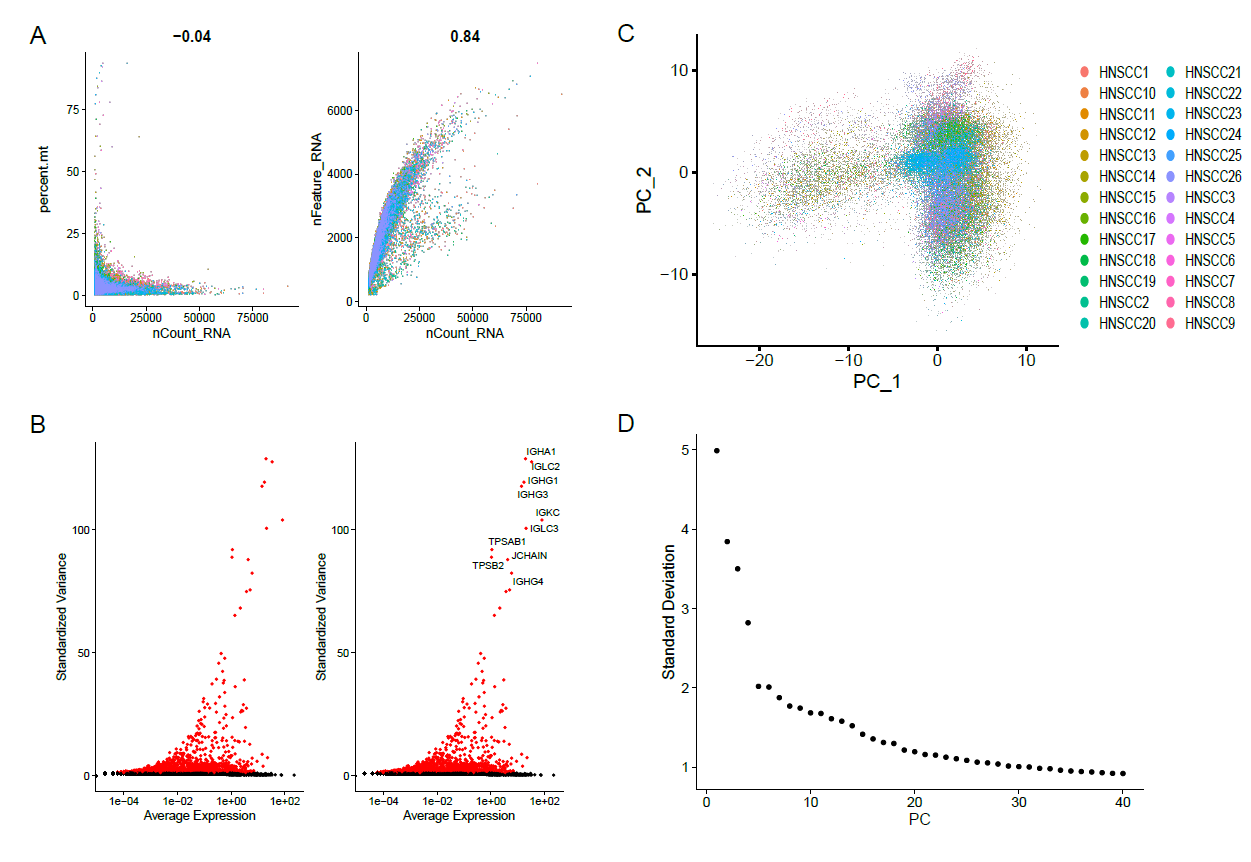


**Figure S1.** Single-cell sequencing data preprocessing process. (A) Relationship between gene number, mitochondrial gene proportion, and UMI number. (B) Genes with highly variable intercellular expression. (C) Principal component analysis dimension reduction showed no obvious batch effect among different samples. (D) Screen plot shows top 40 principal components of principle component analysis. Top 30 principal components were used in downstream analysis.


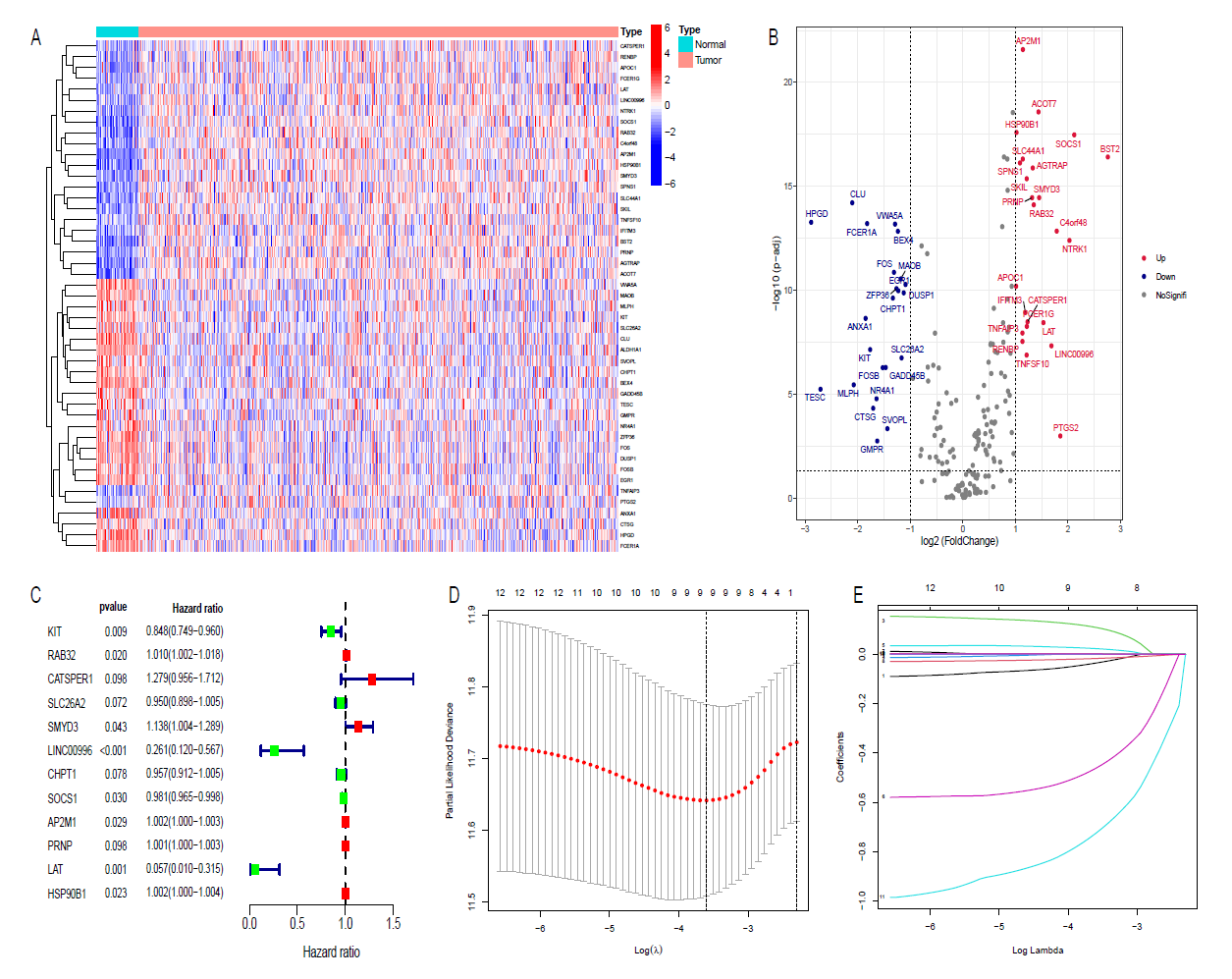


**Figure S2.** Construction of prognostic model with 9 prognostic mast cell characteristic genes in head and neck squamous cell carcinoma. (A–B) Heat maps and volcano plots of differentially expressed mast cell genes (MCGs) between normal tissue and tumor tissue. (C) Forest plots showing MCGs associated with prognosis as determined by univariate Cox regression analysis. (D–E) Lasso regression analysis and partial likelihood deviance were applied to identify 9 prognosis-associated MCGs in the training group.


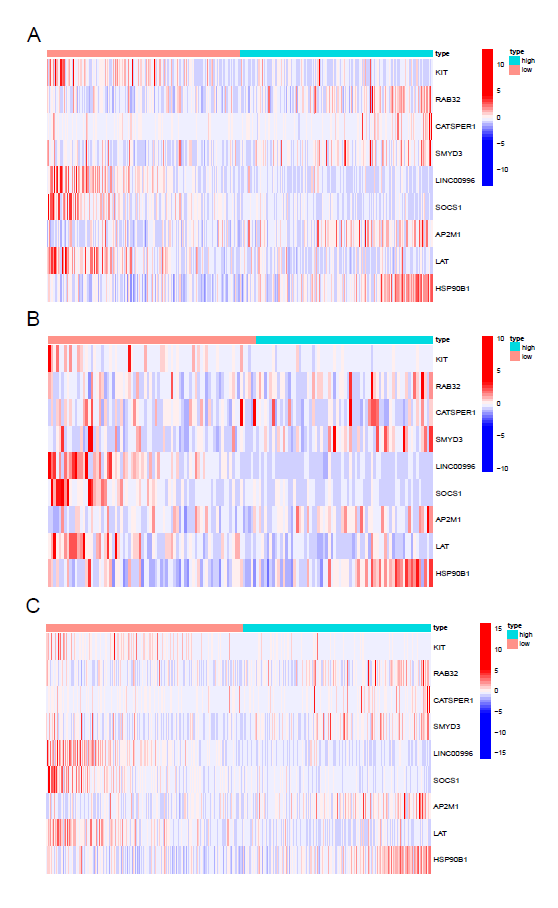


**Figure S3.** Heatmap of differently expressed mast cell signature genes between low- and high risk groups. Training (A), testing (B), and entire (C) cohorts.


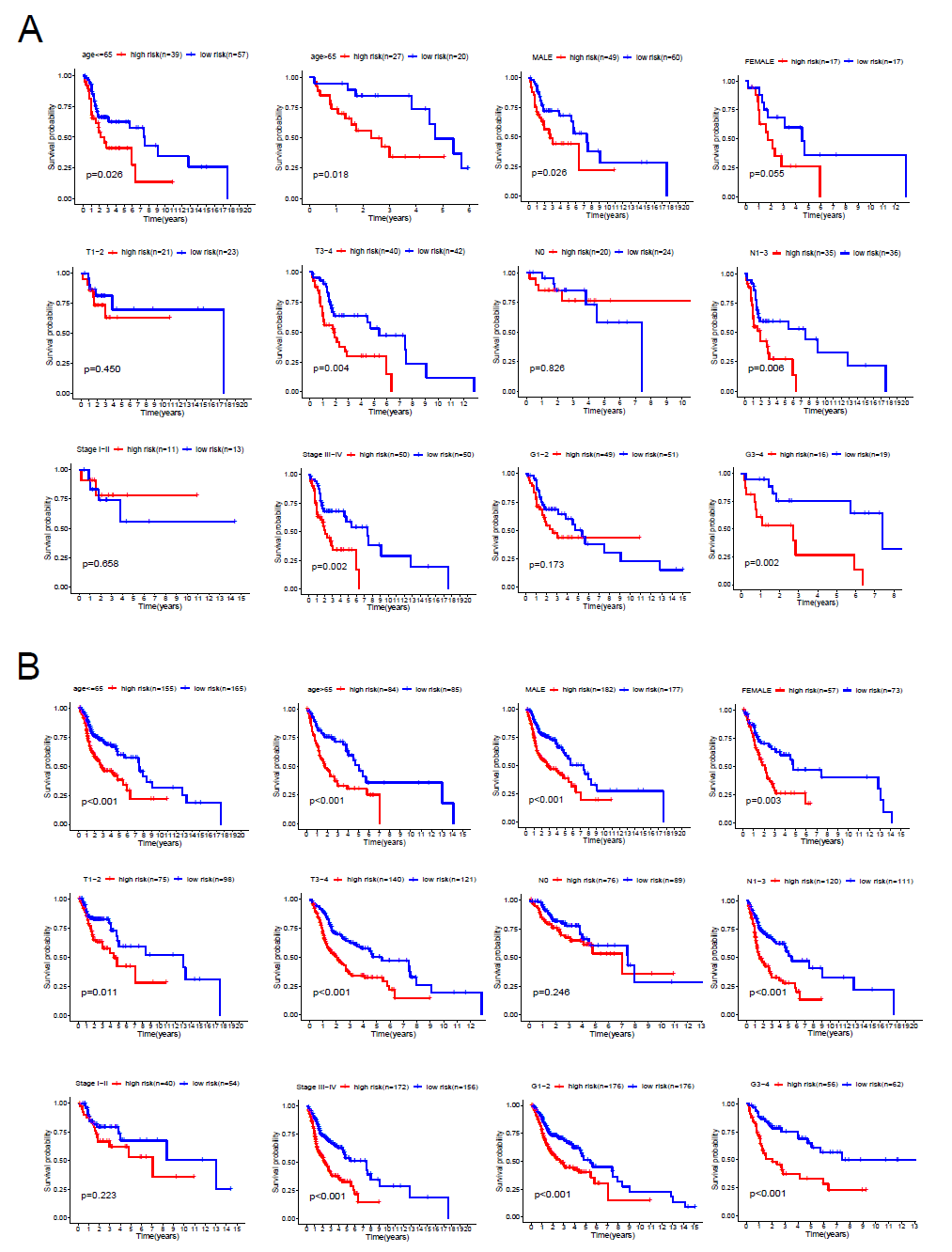


**Figure S4.** Correlation of mast cell gene signature (MCS) risk score with classical clinical variables in the testing (A) and entire TCGA (B) cohorts.


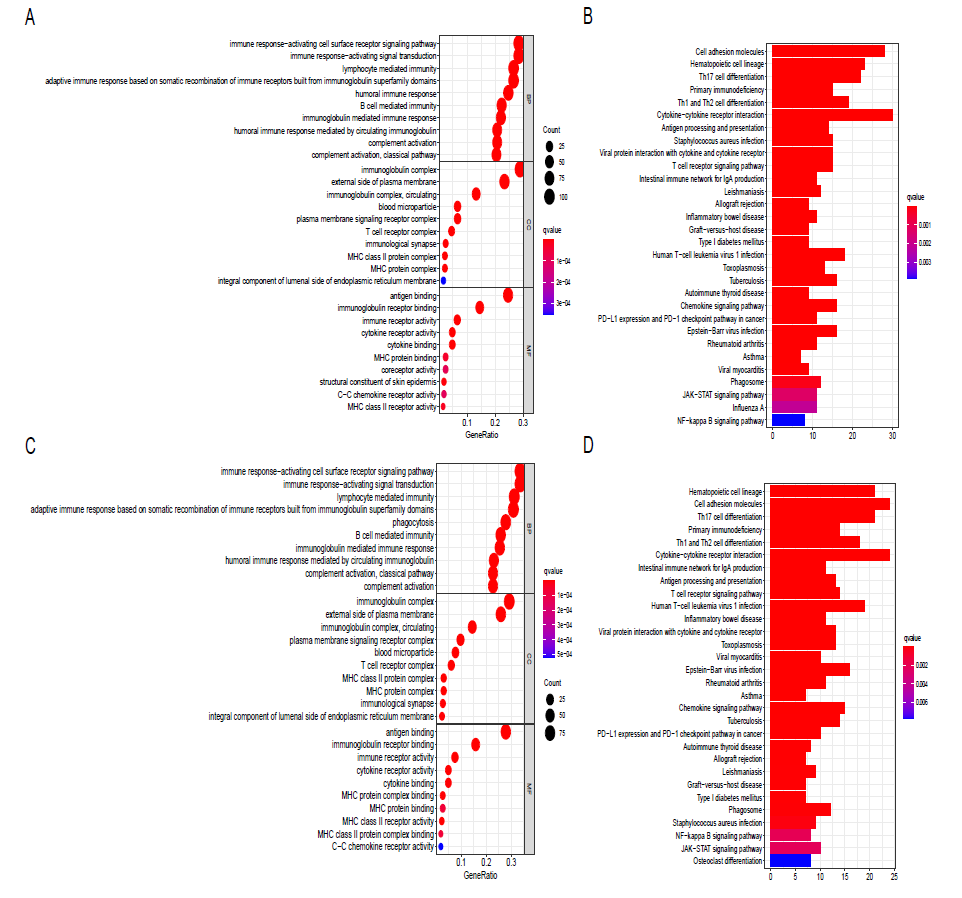


**Figure S5.** Functional enrichment analysis of differentially expressed genes between the two risk groups in the training (A, B) and testing (C, D) cohorts. (A, C) Bubble graph for Gene Ontology enrichment (a larger bubble indicates more enriched genes, and an increasing depth of red indicates greater differences; q-value: adjusted P-value). (B, D) Barplot graph for Kyoto Encyclopedia of Genes and Genomes pathways (longer bar indicates more enriched genes, and an increasing depth of red indicates greater differences).


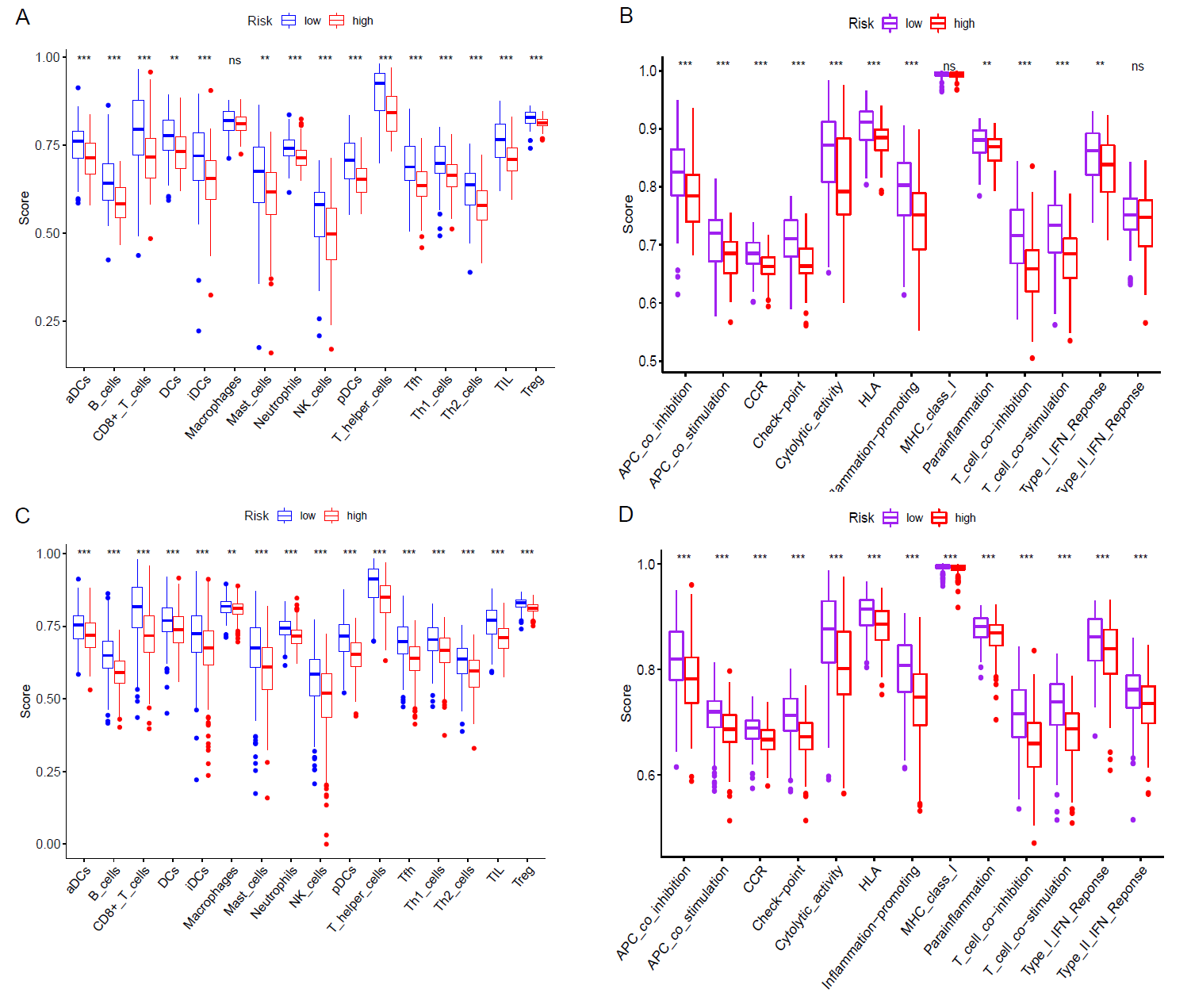


**Figure S6.** Comparison of immune cell infiltration and immune pathways between different risk groups in the entire (A, B) and testing (C, D) The Cancer Genome Atlas cohorts. Comparison of single-set Gene Set Enrichment Analysis scores for 16 immune cell types (A, C) and 13 immune-related functions (B, D) are displayed in boxplots. CCR, cytokine-cytokine receptor. Adjusted P values are shown as: *P < 0.05; ***P < 0.001.


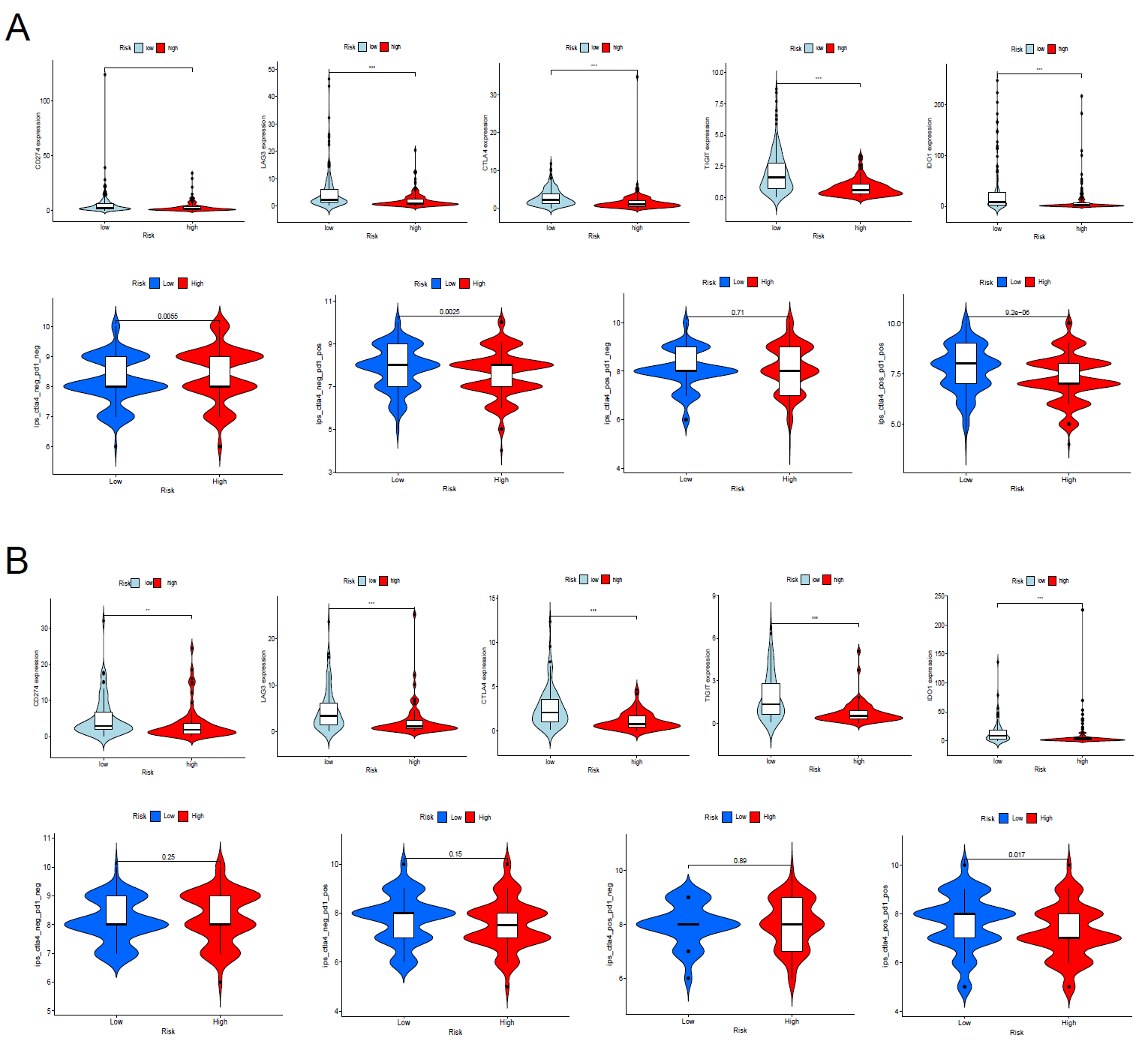


**Figure S7.** Estimation of the role of the mast cell gene signature (MCS) in predicting immune checkpoint gene expression and immunotherapeutic response. Immune checkpoint gene expression in different risk groups of the entire (A) and testing (B) The Cancer Genome Atlas cohorts.

**Table S1.** Clinical information of HNSCC tissues

|  | Immunotherapy works | Immunotherapy fails |
| --- | --- | --- |
| Range of age (year) | 65~75 | 67~68 |
| Grade [n (%)] |  |  |
| Low | 1(50%) | 0 |
| Intermediate | 1(50%) | 1(50%) |
| High | 0 | 1(50%) |
| Tumor site [n (%)] |  |  |
| Hypopharynx | 1(50%) | 2(100%） |
| Hypopharynx+ upper esophagus | 1(50%) | 0 |
| Therapy [n (%)] |  |  |
| Immunotherapy followed failure of initial chemotherapy [n (%)] | 1(50%) | 2(100%） |
| Concurrent chemotherapy and immunotherapy [n (%)] | 1(50%) | 0 |
| Immunotherapeutic effect |  |  |
| Partial response | 2(100%） | 0 |
| Stable disease | 0 | 2(100%） |

**Table S2.** Probe sequences of model genes

| Gene | Probe sequences |
| --- | --- |
| Kit | CAGCGGACCAGCGTGTCGTTGTCTTCTTTC |
| linc00996 | GATCCTTCCGCTTCTTACGCTGCCAACTGC |
| SOCS1 | CGGTAATCGGCGTGCGAACGGAATGTGC |
| LAT | CCTCACAGGCTGGTTCCTCGTTCTCGTAGCT |
| RAB32 | GGGATAGGGCTGCCATTTGGAAGATGAACTTTACT |
| CATSPER1 | GGTGTGGGGGTGATGAGTGAGAGCGAAAGAAC |
| SMYD3 | CTTTTAAGGCATTTGCATTCCCGCTTGTGG |
| AP2M1 | GAGGGCGCGAATGGCACCTCAAAGTTCAT |
| HSP90B1 | TCAAGGGGAGATCATCTGAGTCCACCACAC |

**Table S3.** Expression of Mast cell signature genes in the mast cell cluster

| gene | pct.1 | pct.2 | avg_logFC | FDR |
| --- | --- | --- | --- | --- |
| KIT | 0.667 | 0.001 | 1.415134 | 0 |
| RAB32 | 0.532 | 0.03 | 1.042402 | 0 |
| CATSPER1 | 0.409 | 0.015 | 0.839794 | 0 |
| SMYD3 | 0.484 | 0.047 | 0.900441 | 2.87E-226 |
| LINC00996 | 0.325 | 0.031 | 0.746044 | 1.27E-145 |
| SOCS1 | 0.556 | 0.245 | 0.81305 | 7.39E-31 |
| AP2M1 | 0.694 | 0.401 | 0.52236 | 7.11E-25 |
| LAT | 0.694 | 0.435 | 0.425094 | 3.44E-12 |
| HSP90B1 | 0.567 | 0.38 | 0.520756 | 8.80E-10 |

pct.1: Percentage of cells in the first group that detected this feature (the first group in this table is the mast cell cluster); pct.2: Percentage of cells in the other groups that detected this feature (the other groups in this table include T cells, natural killer cells, B cells, dendritic cells and myeloid cells); avg_logFC: Average log_2_ fold-change between the two groups: positive representation characteristics were expressed higher in the first group; FDR: Adjusted p-value (Nonparametric Wilcoxon rank sum test).
